# Identification of PANoptosis-related genes as biomarkers in ischemic stroke

**DOI:** 10.3389/fneur.2025.1560514

**Published:** 2025-07-25

**Authors:** Anna Jiang, Hongjing Zhang, Xinglei Jia, Huangying Zhao, Hong Zhao, Zhengyu Lu

**Affiliations:** ^1^Department of Neurology, Shuguang Hospital Affiliated to Shanghai University of Traditional Chinese Medicine, Shanghai, China; ^2^Department of Intensive Care Unit, Yueyang Hospital of Integrated Traditional Chinese and Western Medicine, Shanghai University of Traditional Chinese Medicine, Shanghai, China; ^3^Teaching Affairs Department, Yueyang Hospital of Integrated Traditional Chinese and Western Medicine, Shanghai University of Traditional Chinese Medicine, Shanghai, China

**Keywords:** ischemic stroke, neuroinflammation, diagnostic model, PANoptosis, biomarker, machine learning

## Abstract

**Introduction:**

PANoptosis (panoptotic cell death) is an inflammatory, lytic cell death pathway driven by caspases and RIPKs and regulated by PANoptosome complexes, distinguishing it from other cell death pathways. There is a close potential link between PANoptosis and neuroinflammation, with both regulating each other through complex molecular mechanisms and jointly participating in the pathological processes of neurological diseases.

**Methods:**

To investigate whether PANoptosis exists in IS and identify the master regulators of PANoptosis and their relationship. Gene microarray data were downloaded from the Gene Expression Omnibus (GEO) and differentially expressed genes (DEGs) were identified using R software. R software and Cytoscape were used to analyze and visualize the data. Gene ontology-biological process and the Kyoto Encyclopedia of Genes and Genomes were used to analyze the biological processes and possible pathways. The LASSO regression analysis, Random Forest (RF) and support vector machine (SVM) methods were used to identify key genes for diagnostic model construction. In addition, biomarkers with higher diagnostic values for ischemic stroke were validated using other GEO datasets.

**Results and discussion:**

Finally, 4,392 upregulated genes and 4,356 downregulated genes were identified in the peripheral blood of 23 normal controls and 69 patients with IS from the GSE58294 dataset. Crossing the differential genes with 277 PANoptosis genes yielded 60 upregulated genes and 58 downregulated genes. The top 10 hub upregulated genes and hub downregulated genes were identified using Cytoscape. Through LASSO regression, RF and SVM, four intersecting genes were screened from upregulated genes, and six intersecting genes were screened from downregulated intersecting genes. These ten intersecting genes were differentially expressed in the validation GSE16561 dataset. The results identify upregulated genes (CASP1, CTNNB1, CASP8) and downregulated genes (PSMC3) as key regulators of PANoptosis in IS. These findings demonstrate that PANoptosis-related genes are differentially expressed in IS and may serve as potential biomarkers.

## 1 Introduction

With the aging of the world’s population, stroke has become a serious health problem worldwide and deserves our attention (1). Ischemic Stroke (IS) is the most common cerebrovascular event (2). IS is a group of clinical syndromes caused by various blood supply disorders in a specific brain region, leading to hypoxic–ischemic necrosis and neurological dysfunction. According to the TOAST classification (3) and related studies, the causes of ischemic cerebrovascular disease include cardiogenic embolism, small-artery occlusion, and cerebral atherosclerosis. IS has a high incidence and accounts for approximately 80% of all cerebrovascular diseases (4). In addition, it has the characteristics of high mortality, disability rate, and recurrence rate.

Neuroinflammation in IS is the main pathological event in ischemic cerebrovascular disease. IS can cause further necrosis and apoptosis of nerve cells, and necrosis of nerve cells leads to the release of cytokines, which further initiates the neuroinflammatory response and promotes cell death. Cell death is a widespread and basic physiological mechanism for the body to maintain its stability. With the development of cell death research, programmed cell death (PCD) has been proposed in recent decades. Of the proposed forms of PCD, pyroptosis, apoptosis, and necroptosis are the most clearly defined. Their molecular mechanisms are complex and regulate the initiation, transduction, and execution of cell death (5, 6). All three PCDs are associated with IS (7). Apoptosis occurs due to the overexpression of caspase-1, 3, 6, 8, and 9 in the infarct core after IS. Pyroptosis is induced by the production of inflammasomes, such as NOD-like receptor thermal protein domain associated protein 3 (NLRP3), via microglial activation after IS (8). IS necroptosis is caused by the activation of receptor-interacting serine/threonine-protein kinase 1 (RIPK1) and the phosphorylation of receptor-interacting serine/threonine-protein kinase 3 (RIPK3) and mixed-lineage kinase domain-like (MLKL) caused by increased tumor necrosis factor alpha (TNF-*α*) content after IS. These three types of PCDs are closely intertwined with the inflammatory response. Specifically, pyroptosis is driven by inflammatory factors (9, 10), apoptosis is regulated by inflammatory signals (11), and necroptosis is initiated by inflammatory cues (12, 13).

We previously thought that these three PCDs were independent; however, in recent years, an increasing number of studies have found that there are interactions among these three PCDs (14). The integration of the three PCD lines led to a new concept, PANoptosis. PANoptosis is an inflammatory PCD pathway that shares the essential features of the three PCDs but cannot be explained by any of them alone. PANoptosis usually has been identified as an inflammatory, lytic cell death pathway driven by caspases and RIPKs and regulated by PANoptosome complexes, making it distinct from other cell death pathways (15). It also has the key characteristics of three death modes: apoptosis, necrosis and necrotic apoptosis (16). The occurrence of extensive apoptotic PCD requires the drive of a complex called the PANoptosome, which contains crucial molecules in pyroptosis, apoptosis, and necroptosis (17). PANoptosome has been proved to contain RIPK1, apoptosis-associated speck-like protein (ASC), NLRP3, Caspase-8 (CASP8), RIPK3, CASP6, Z-DNA binding protein 1 (ZBP1), CASP1, etc. (18, 19). When cells are exposed to stimuli such as pathogen infection, oxidative stress, or ischemia-hypoxia, the PANoptosis signaling pathway is activated. For example, during viral infection, viral nucleic acids are recognized by ZBP1, which interacts with RIPK1 and RIPK3 to promote PANoptosome assembly. In normal conditions, CASP8 in the PANoptosome initiates apoptosis. However, when CASP8 activity is inhibited, RIPK1 and RIPK3 phosphorylate MLKL, leading to MLKL oligomerization and pore formation in the cell membrane, triggering necroptosis. Meanwhile, activation of the NLRP3 inflammasome leads to CASP1 activation, which cleaves Gasdermin D to form active fragments that create pores in the cell membrane, causing cell swelling, rupture, and release of inflammatory factors, thereby inducing pyroptosis. Throughout this process, signaling pathways related to apoptosis, necroptosis, and pyroptosis are intricately intertwined and synergistically drive cells toward PANoptosis (6, 15, 19, 20). However, the activation of PANoptosome is triggered by cell death and the inflammatory response after IS (21). Studies have shown that PANoptosis is likely to exist in nervous system diseases or injuries in addition to infectious diseases (22). The study found that in cellular models simulating ischemic brain injury, such as different passaged cell lines (PC12 cells, SH-SY5Y cells) and various primary neurons (primary hippocampal cells, primary cortical cells), pyroptosis, apoptosis, and programmed necrosis coexist. Evidence of pyroptosis, apoptosis, and programmed necrosis has also been observed in rat and mouse models of cerebral ischemia–reperfusion injury. These results indicate that PANoptosis occurs in experimental ischemic brain injury, suggesting that PANoptosis may be involved in the regulation of various central nervous system diseases (22). However, PANoptosis has not been characterized in human stroke or experimental *in vivo*/*in vitro* models. Moreover, research on PANoptosis provides new perspectives for the study of neurological diseases. Traditionally, studies on cell death in neurological diseases have often treated pyroptosis, apoptosis, and programmed necrosis as independent processes. However, the concept of PANoptosis suggests that these different forms of cell death may be interconnected in a complex regulatory network, collectively contributing to disease development. Taking ischemic stroke as an example, elucidating the mechanisms of PANoptosis could enhance our understanding of the pathophysiology of ischemic brain injury and provide a theoretical basis for developing novel therapeutic targets and strategies. For instance, identifying key molecules and signaling pathways in PANoptosis may enable interventions to alleviate ischemic brain damage, protect neurons, and improve patient outcomes (22–24).

In this study, we analyzed GSE58294 gene data to identify DEGs in peripheral blood samples from IS patients and healthy controls. By analyzing the intersection of upregulated and downregulated DEGs and PANoptosis-related genes, we identified intersecting genes that may be involved in the regulation of PANoptosis in IS. To further analyze the main biological functions and possible pathways regulated by the differentially expressed genes and intersecting genes, biological process (BP) and Kyoto Encyclopedia of Genes and Genomes (KEGG) enrichment analyses were performed. Next, we explored the interactions between intersecting genes by protein–protein interaction (PPI) network construction and extracted 10 hub genes at the centers of up- and downregulated intersecting genes using Cytohubba in Cytoscape. Finally, the diagnostic model was constructed by lasso regression analysis, the receiver operating characteristic (ROC) curve was used to analyze the efficacy of the diagnostic model, and the diagnostic model was validated again on the validation dataset. In this study, we explored the regulation of PANoptosis-related genes in IS and analyzed the possible pathways of action for PANoptosis in IS. The construction and validation of the diagnostic model suggest that genes associated with PANoptosis may be significant biomarkers in IS, which provides a current reference for further study of the role of PANoptosis in IS.

## 2 Materials and methods

### 2.1 Gene expression profile data

The expression data for IS were downloaded from the GEO (Gene Expression Omnibus)^1^ database (GSE58294), and the DEGs associated with IS were screened. GSE58294 was selected as the core dataset for its rational sample size, disease relevance, reliable data quality, and analytical applicability. A total of 92 samples were included from the GSE58294 dataset, including 69 IS cases and 23 normal controls. Gene expression was detected by using GPL570 [HG-U133_Plus_2] Affymetrix Human Genome U133 Plus 2.0.

### 2.2 PANoptosis gene list

PANoptosis is composed of genes, 27 from pyroptosis, 242 from apoptosis, and eight from necroptosis; the genes for PANoptosis were taken from the literature (22, 25). In this study, the gene table of PANoptosis was generated by combining the gene tables of pyroptosis, apoptosis, and necrosis and removing overlapping genes (Supplement 1).

### 2.3 Identification of differentially expressed genes with R software

The distribution of the two samples was observed using Principal Component Analysis (PCA). Data normalization was performed on the GSE58294 dataset using the ‘limma’ (26) and other packages of R software (version 3.6.3) and derived DEGs between normal and IS samples with DEG screening criteria of adjusted *p*-value < 0.05, and | log fold change (FC) | > 0. The upregulated and downregulated DEGs were crossed with PANoptosis genes. A Venn diagram was used to show the crossover between DEGs (27).

### 2.4 Functional enrichment analysis of DEGs

The upregulated and downregulated DEGs were analyzed using the enrichGO function in the ‘clusterProfiler’ package, and enrichKEGG analysis was performed using the enrichKEGG function. We then performed BP and KEGG analyses of the upregulated and downregulated intersecting genes. DEG and GO/KEGG analyses provide “statistical evidence” and “functional annotations” for biomarkers, ensuring their relevance to the PANoptosis pathway.

### 2.5 PPI network construction and identification of hub genes

Based on the identified upregulated and downregulated intersecting genes, a PPI network was constructed using the Interactive Gene database retrieval tool (STRING)^2^. The minimum required interaction score was set to medium confidence (0.4), and the species was limited to *Homo sapiens*. The identified upregulated and downregulated intersecting genes were visualized using Cytoscape3.9.0 (28) and 10 upregulated and downregulated hub genes were extracted using CytoHubba. CytoHubba, a plugin of Cytoscape software that measures nodes based on their network characteristics and thus allows the exploration of important nodes in biological networks, was used to identify hub genes (29). PPI and Hub gene analysis reveal the core position of biomarkers in molecular networks, explaining their potential as therapeutic targets. Screening of hub genes was implemented via the Cytoscape plugin CytoHubba, based on the connectivity (Degree) of molecular interaction networks. Genes with extensive interactions were prioritized to ensure their structural importance in the PANoptosome.

### 2.6 Lasso regression, random forest, and the support vector machine

LASSO regression, Random Forest (RF), and Support Vector Machine (SVM) were performed in R to screen final hub genes and construct diagnostic models (30). Random forest is an ensemble learning method that makes predictions by training multiple decision trees simultaneously. In genetic screens, random forest can help us identify the genes that impact the most on the target variable (such as disease status). By looking at the importance score of each feature (gene) in the random forest model, we can determine what genes are the most critical for distinguishing between different sample categories. The support vector machine is a supervised learning model with relevant learning algorithms for analyzing data for classification and regression analysis. By screening characteristic genes by three machine learning methods, and finally cross the final key genes. After gene selection using LASSO, RF and SVM were used to build models separately, and the average probability was taken as the final output to reduce the risk of overfitting in single models.

### 2.7 Construction of diagnostic model

**Figure 1:**
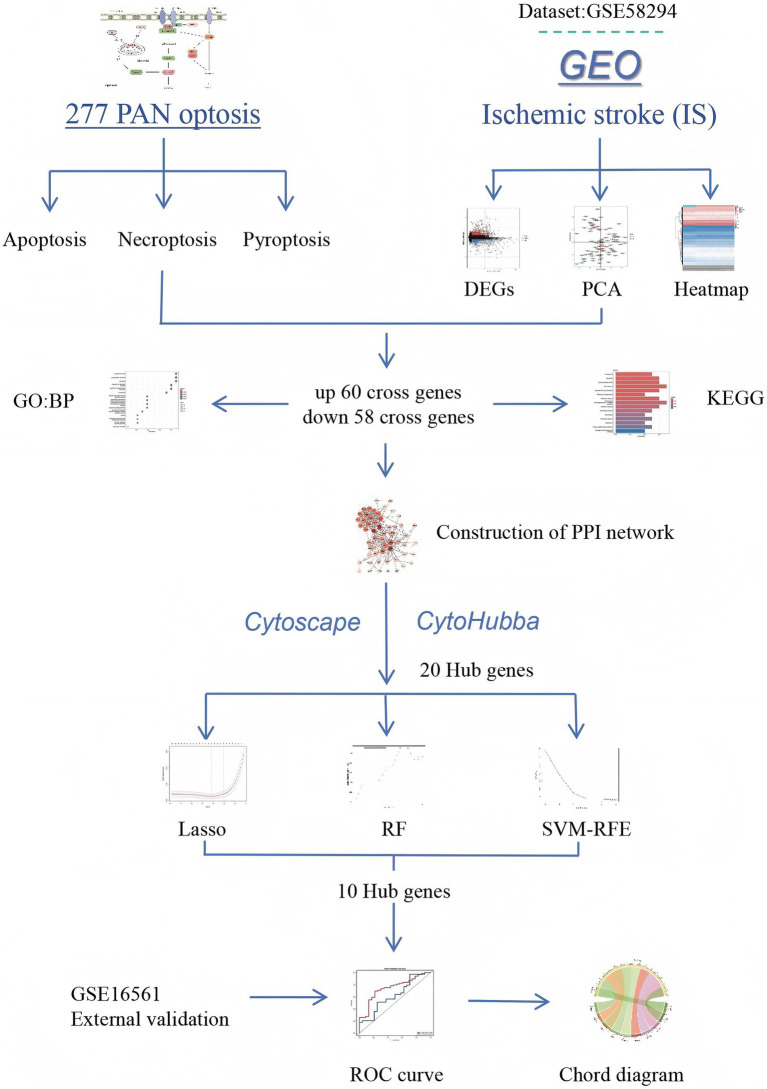
Flowchart.

ROC curves were plotted by using the pROC package in the R language to evaluate the prediction accuracy of the diagnostic models (31), and the performance of the key genes in predicting disease samples was evaluated by plotting the ROC curves of the key genes. Finally, to validate the reliability of the diagnostic model, the GSE16561 dataset was selected from the GEO database to verify the diagnostic value of the diagnostic model. To quantify the accuracy of the diagnostic model, the closer the area under curve (AUC) value is to 1, the larger the AUC, indicating a higher accuracy of the diagnostic model. Otherwise, the accuracy of the diagnostic model decreases. If the curve is closer to the upper-left corner, the abscissa is smaller and the ordinate is larger, indicating that the diagnostic model is more accurate. ROC curves evaluate the clinical translation value of biomarkers from the perspective of “prediction accuracy,” providing a basis for subsequent prospective validation. Through the organic integration of the above methods, the study systematically identified PANoptosis-related biomarkers in IS, offering new directions for the early diagnosis and mechanistic research of ischemic stroke (Figure 1).

## 3 Results

### 3.1 Identification of DEGs

**Figure 2:**
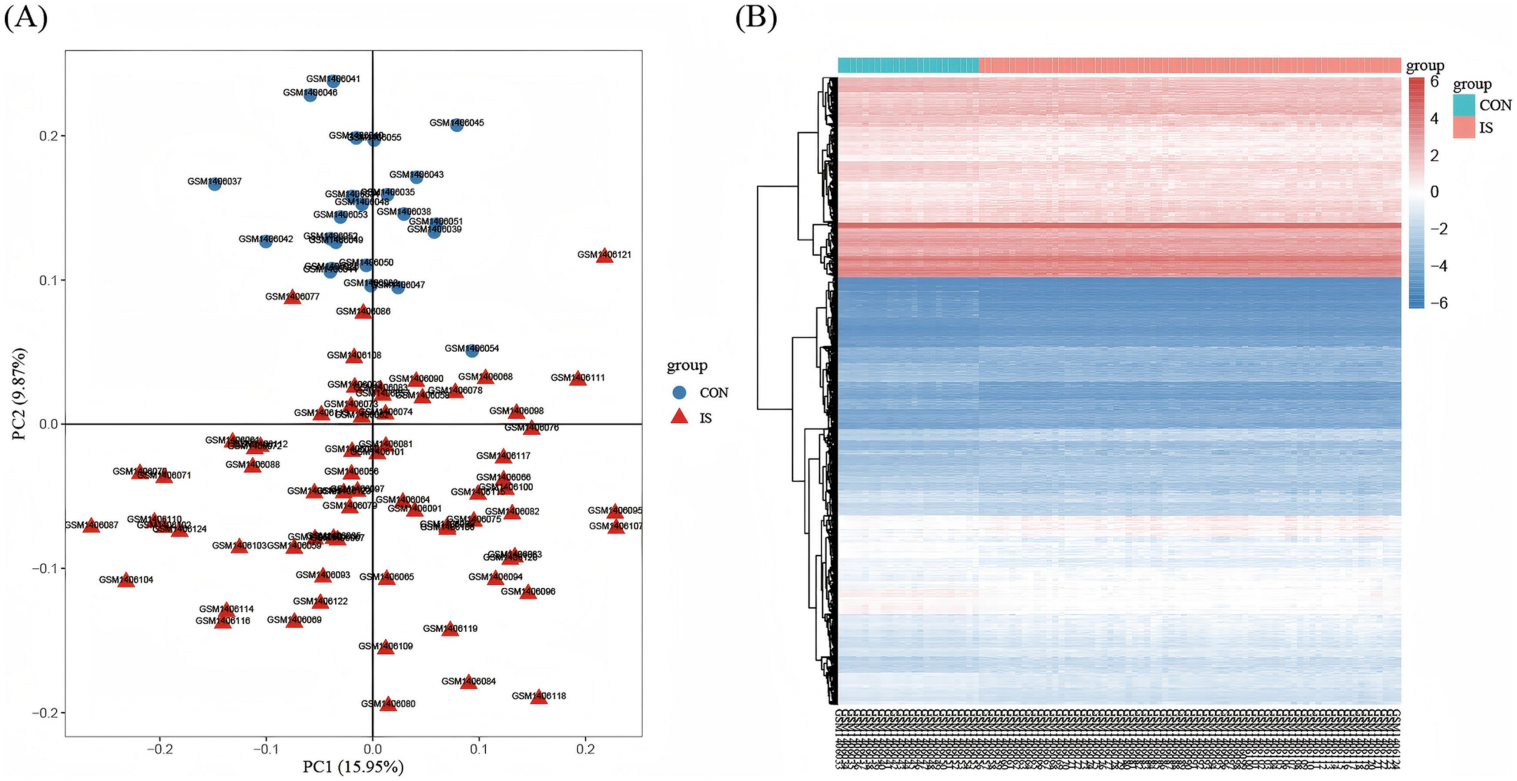
Identification of DEGs. **(A)** PCA shows the distribution between the two samples. The red triangle icon represents the disease group sample, and the blue circle icon represents the normal group sample. The abscissa represents principal component 1 with a variance contribution rate of 15.95%, and the ordinate represents principal component 2 with a variance contribution rate of 9.87%. **(B)** Heatmap of DEGs between IS samples and CON samples, where blue represents down-regulation and red represents up-regulation. The abscissa shows 92 samples, the first 23 of which are normal group samples, which are represented in blue; The last 69 samples are disease group samples, which are indicated in red. The ordinate represents the distribution of all differential genes in the sample. Adjusted *p*-value < 0.05, and | log fold change (FC) | ≥ 0.

**Figure 3:**
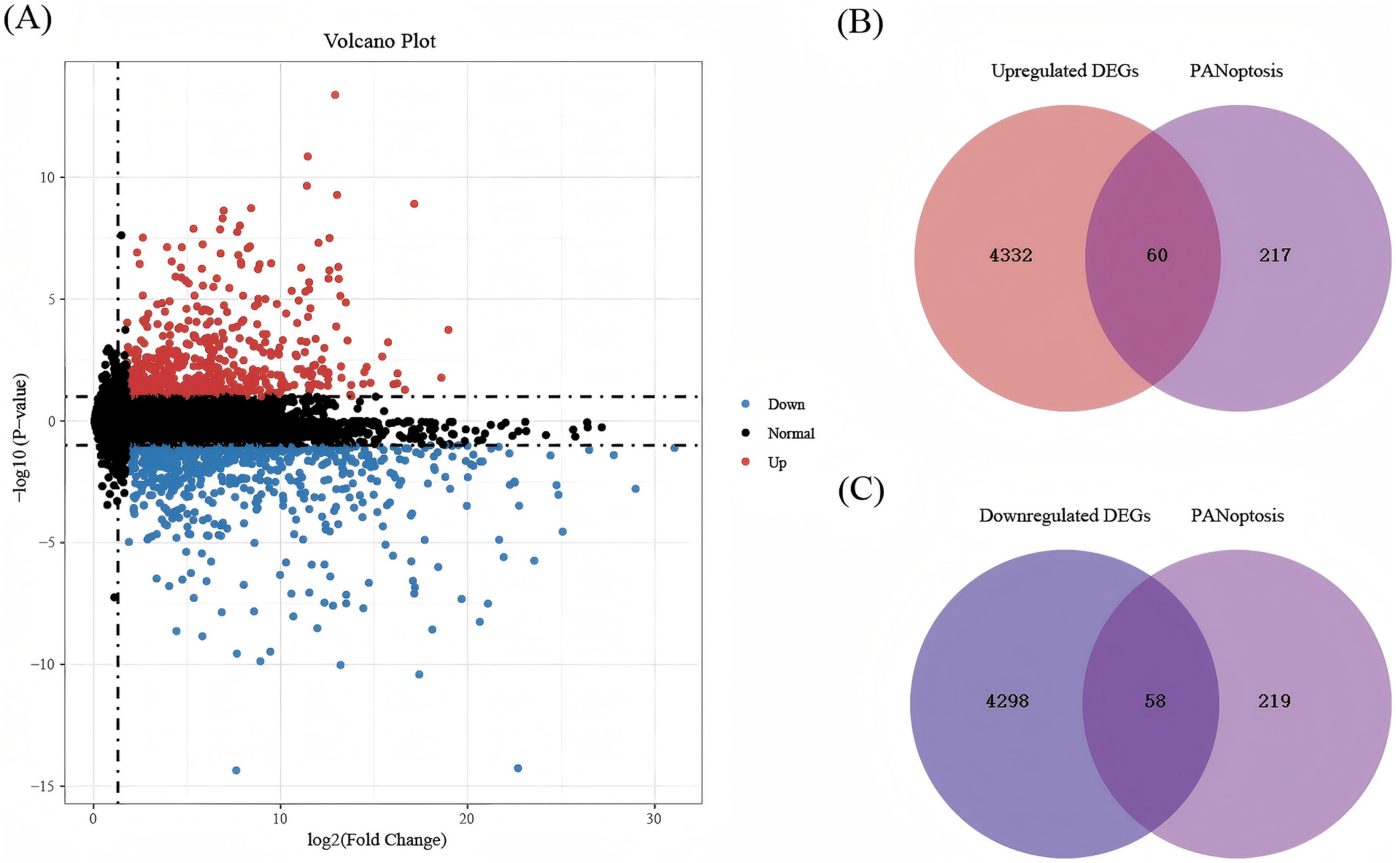
The volcanic plot and Venn diagrams. **(A)** The volcanic plot between IS and healthy samples in GSE58294. Blue indicates significant downregulation, red indicates significant upregulation, and black indicates insignificant DEGs. The abscissa represents the corrected *p*-value after the -log10 transformation and the ordinate represents the difference multiple. **(B)** Venn diagrams regarding the intersection between upregulated DEGs and PANoptosis genes. **(C)** Venn diagrams regarding the intersection between downregulated DEGs and PANoptosis genes.

In GSE58294, the differentially expressed genes were identified in the peripheral blood of 23 controls and 69 patients with IS, including 4,392 upregulated genes and 4,356 downregulated genes (adjusted *p* < 0.05). PCA was used to assess sample distribution and separation, revealing a subtle but discernible distinction between the two groups (Figure 2A). Second, we visualized the expression of the most significant DEGs across all samples via a heat map, in which blue represents downregulation and red represents up-regulation (Figure 2B). As shown in Figure 3A, we plotted all the DEGs with a volcano diagram; DEGs with log2FC < −1 are indicated in blue, and those with log2FC > 1 are indicated in red. Subsequently, 60 intersecting genes were obtained based on the intersection of upregulated DEGs and PANoptosis genes, and 58 intersecting genes were obtained based on the intersection of downregulated DEGs and PANoptosis genes (Figures 3B,C).

### 3.2 Functional enrichment analysis of DEGs

**Figure 4:**
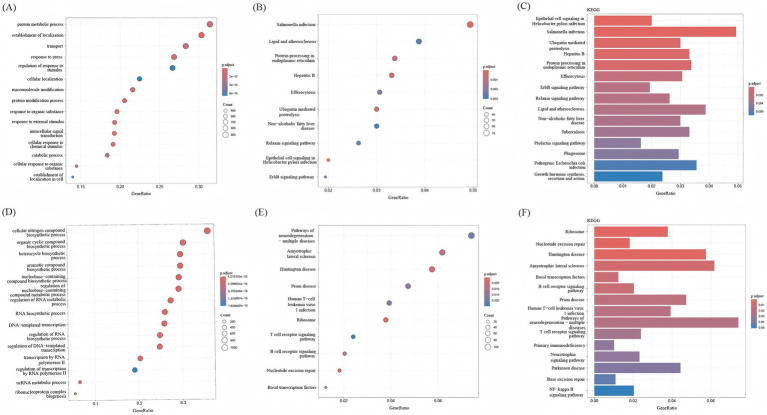
KEGG analysis and BP analysis of DEGs. **(A)** BP analysis of upregulated DEGs. **(B)** Bar graphs show KEGG enrichment analysis of downregulated DEGs, with increasing enrichment from small to large adjusted *p*-values of different colors represented from blue to red. **(C)** Bubble plots showed KEGG enrichment analysis of upregulated DEGs; p.adjust represents enrichment analysis significance, and the adjusted p-values from blue to red represent increasing enrichment for different colors from small to large. **(D)** BP analysis of downregulated DEGs. **(E)** Bar graphs show KEGG enrichment analysis of downregulated DEGs, with increasing enrichment from small to large adjusted p-values of different colors represented from blue to red. **(F)** Bubble plots showed KEGG enrichment analysis of downregulated DEGs; p.adjust represents enrichment analysis significance, and the adjusted *p*-values from blue to red represent increasing enrichment for different colors from small to large.

BP analysis of the overall upregulated and downregulated DEGs was performed using the enrichGo function in the ‘clusterProfiler’ package, and enrichKEGG enrichment analysis was performed by using enrichKEGG. Through BP analysis, the upregulated DEGs were mainly related to protein metabolic processes, the establishment of localization, transport, response to stress, and other biological processes (Figure 4A). The downregulated DEGs were mainly related to cellular nitrogen compound biosynthetic processes and other biological processes (Figure 4D). According to the KEGG bubble diagram and bar diagram, upregulated DEGs were mainly enriched in Salmonella infection, Protein processing in the endoplasmic reticulum, Hepatitis B, Ubiquitin mediated proteolysis, etc. (Figures 4B,C). The downregulated DEGs were mainly enriched in Huntington disease, Ribosome, Nucleotide excision repair, Amyotrophic lateral sclerosis, etc. (Figures 4E,F).

### 3.3 Functional enrichment analysis of intersecting genes

**Figure 5:**
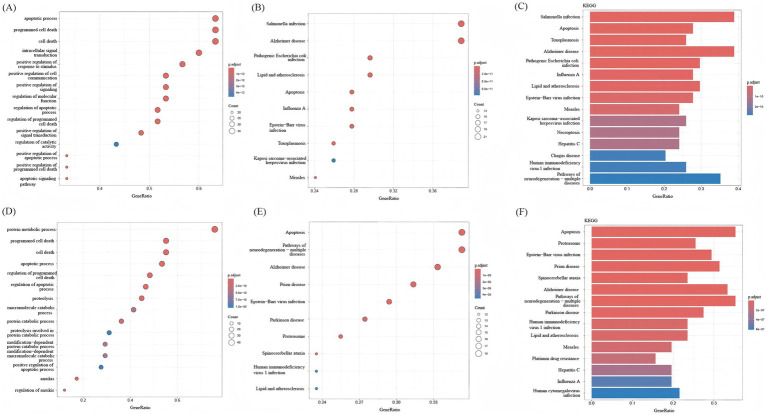
KEGG analysis and BP analysis of intersecting genes. **(A)** BP analysis of upregulated intersecting genes. **(B)** The bar graph shows KEGG enrichment analysis of upregulated intersecting genes, with increasing enrichment from smaller to larger adjusted *p*-values for different colors from yellow to red. **(C)** The bubble plot shows KEGG enrichment analysis of upregulated intersecting genes, with increasing enrichment from smaller to larger adjusted *p*-values for different colors from yellow to red. **(D)** BP analysis of downregulated intersecting genes. **(E)** The bar graph shows KEGG enrichment analysis of downregulated intersecting genes, with increasing enrichment from smaller to larger adjusted p-values for different colors from yellow to red. **(F)** The bubble plot shows KEGG enrichment analysis of downregulated intersecting genes, with increasing enrichment from smaller to larger adjusted p-values for different colors from yellow to red.

The intersecting genes between PANoptosis and upregulated genes and the intersecting genes between PANoptosis and downregulated genes were analyzed again by BP analysis and KEGG enrichment analysis. Through BP analysis, the upregulated intersecting genes were primarily related to the regulation of the apoptotic process, programmed cell death, cell death, and other biological processes (Figure 5A). The downregulated intersecting genes were primarily related to protein metabolic processes, programmed cell death, cell death, apoptotic process, and other biological processes (Figure 5D). According to the KEGG bubble diagram and bar diagram, the upregulated intersecting genes were mainly enriched in Salmonella infection, Alzheimer disease, Pathogenic *Escherichia coli* infection, lipid and atherosclerosis, and apoptosis (Figures 5B,C). The downregulated intersecting genes were mainly enriched in Apoptosis, Pathways of neurodegeneration-multiple diseases, Alzheimer disease, and Prion disease (Figures 5E,F). The enrichment of upregulated genes in necroptosis highlights RIPK3/MLKL-mediated cell death as a key driver of inflammatory tissue damage in IS. In preclinical models, MLKL inhibition reduces infarct size by 40%, supporting its role in PANoptosis execution (32). Downregulated genes in apoptosis suggest impaired anti-apoptotic signaling (e.g., BCL2L1 downregulation), which may disrupt the balance between physiological and pathological cell death. This aligns with our finding that PSMC3, a proteasomal regulator of BCL2 stability, is downregulated in IS.

### 3.4 PPI network construction and identification of hub genes

**Figure 6:**
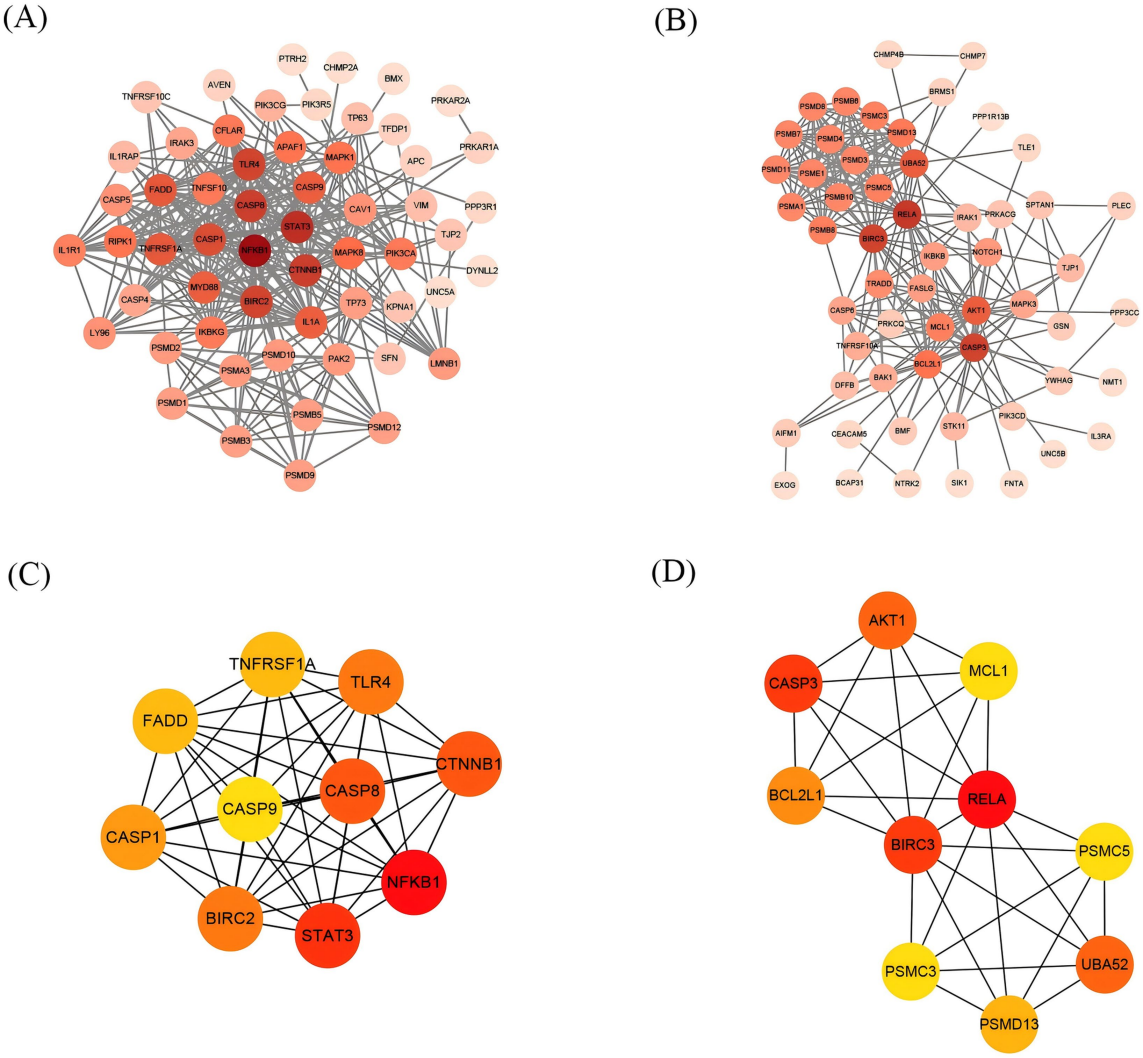
The construction of the PPI network and Screening of Hub Genes. Each origin represents a gene, and the darker the color, the more genes intersect with it. **(A)** PPI network of upregulated intersecting genes. **(B)** PPI network of downregulated intersecting genes. **(C)** The top 10 gene network maps of the related upregulated intersecting genes were screened by degree. **(D)** The top 10 gene network maps of the related downregulated intersecting genes were screened by degree, with darker colors from yellow to red indicating a greater degree of correlation.

Based on the identified upregulated and downregulated intersecting genes, a PPI network was constructed by using the Interactive Gene database retrieval tool (STRING) (see text footnote 2) (Figures 6A,B). Using the CytoHubba plugin of Cytoscape3.9.1 software, the top 10 hub genes of the upregulated and downregulated intersecting genes screened, respectively, are shown in Figures 6C,D. From the perspective of biological interaction, the top 10 hub genes were selected for the following machine-learning analysis.

### 3.5 LASSO regression

**Figure 7:**
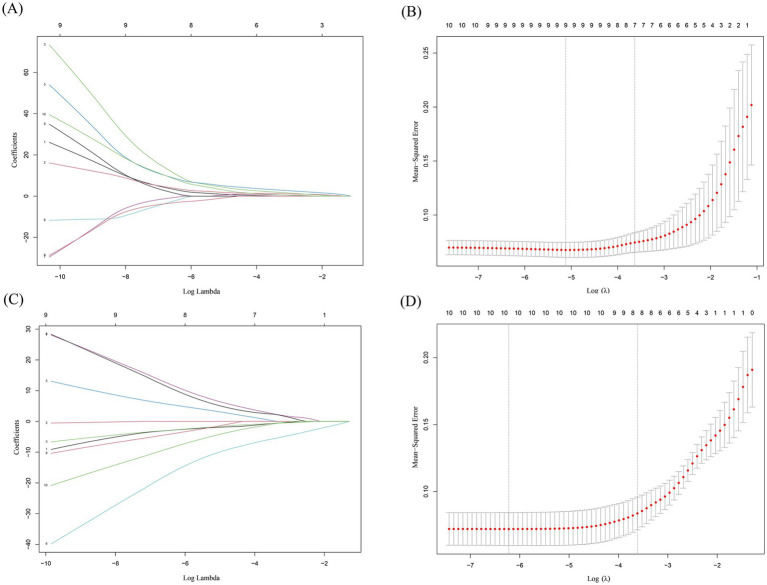
Lasso regression analysis results. **(A,C)** This plot illustrates how the coefficients of different features (genes) change as the regularization parameter *λ* (represented on the x - axis as Log Lambda) varies in a LASSO (Least Absolute Shrinkage and Selection Operator) regression model. Each line corresponds to a different feature, and the y - axis shows the coefficient values. As λ increases, some coefficients shrink toward zero, indicating that those features are being penalized more heavily and may be removed from the model. This visualization helps to understand which features are more robust and less likely to be shrunk out as the regularization strength increases. **(B,D)** The x - axis represents the log - transformed regularization parameter λ (Log λ). The y - axis shows the mean squared error, which measures the average squared difference between the predicted and actual values. The red dots represent the MSE values for different λ values, and the shaded area around them likely represents a confidence interval. The vertical dashed lines indicate the optimal λ values selected based on cross - validation. The goal is to find the λ that minimizes the MSE, balancing model complexity and prediction accuracy. Figure **A** and **B** demonstrate results of Lasso regression analysis of 10 upregulated hub intersecting genes. Figure **C** and **D** demonstrate results of Lasso regression analysis of 10 downregulated hub intersecting genes.

LASSO is widely used in gene selection to identify key disease biomarkers. For example, in cancer research, LASSO has successfully selected core gene sets associated with prognosis from thousands of genes (30). Lasso regression analysis was performed on the 10 upregulated hub intersecting genes and 10 downregulated hub intersecting genes to select the genes for the final model. For the 10 upregulated hub intersecting genes, each curve in the lambda plot represents the independent variable trajectory of each independent variable coefficient, with the ordinate representing the value of the coefficient and the abscissa L1 norm (Figure 7A). After selecting the best *λ* value (lambda. min), seven characteristic genes (Figure 7B) and their corresponding correlation coefficients were obtained. For the 10 downregulated hub intersecting genes, through lasso regression analysis and the selection of the best λ value (lambda. min), eight characteristic genes (Figures 7C,D) and their corresponding correlation coefficients were screened. All of these demonstrate the efficiency of LASSO in dimensionality reduction.

### 3.6 Random forest

**Figure 8:**
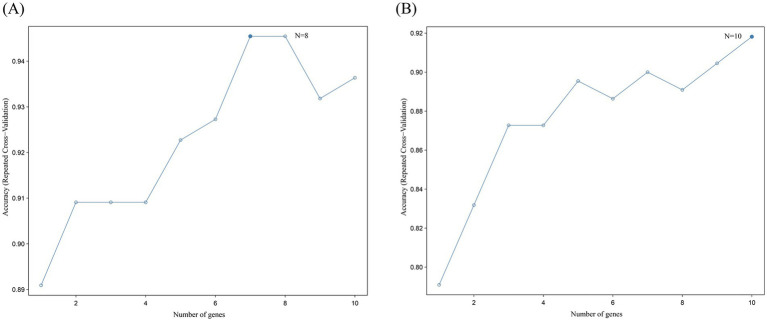
Random forest analysis results. **(A)** Results of random forest analysis of 10 upregulated hub intersecting genes. This line plot depicts the relationship between the number of genes and the accuracy of repeated cross - validation. The x-axis represents the number of genes, ranging from 1 to 10, while the y-axis shows the accuracy of repeated cross-validation. Each data point on the line indicates the accuracy achieved with a specific number of genes. The highest accuracy is reached when the number of genes is 8, as indicated by “*N* = 8,” and the accuracy value at this point is approximately 0.95. This suggests that, within the context of the analysis, using 8 genes results in the best performance in terms of repeated cross-validation accuracy. **(B)** Results of random forest analysis of 10 downregulated hub intersecting genes. This line plot also shows the relationship between the number of genes and the accuracy of repeated cross-validation. The x-axis is the number of genes, and the y-axis is the accuracy of repeated cross-validation. The data points on the line represent the accuracy values corresponding to different numbers of genes. The highest accuracy is obtained when the number of genes is 10, as marked by “*N* = 10,” with an accuracy value close to 0.92. This indicates that, for this particular analysis, a set of 10 genes yields the optimal performance in repeated cross-validation. These plots are crucial for determining the optimal number of genes to use in a predictive model to achieve the best generalization and prediction accuracy through repeated cross-validation.

RF has shown robustness to noisy data in biomarker screening. For example, in Alzheimer’s disease gene analysis, RF successfully identified gene modules associated with *β*-amyloid deposition (33). Random forest was performed on the 10 upregulated hub intersecting genes and 10 downregulated hub intersecting genes to select the genes for the final model. All 10 upregulated genes were subjected to 10-fold cross-validation and the result showed the highest accuracy when eight genes were included (Figure 8A). All 10 downregulated genes were subjected to 10-fold cross-validation and the result showed the highest accuracy when eight genes were included (Figure 8B). Therefore, these eight upregulated genes and 10 downregulated genes can be regarded as maker genes between the normal and disease groups. For example, in this study, RF determined the optimal number of genes as 8 through 10-fold CV (Figure 8A), and the feature importance ranking highly overlapped with LASSO results (e.g., CASP1 and CASP8 were both in the top 3 genes), verifying the reliability of the findings.

### 3.7 Support vector machine

**Figure 9:**
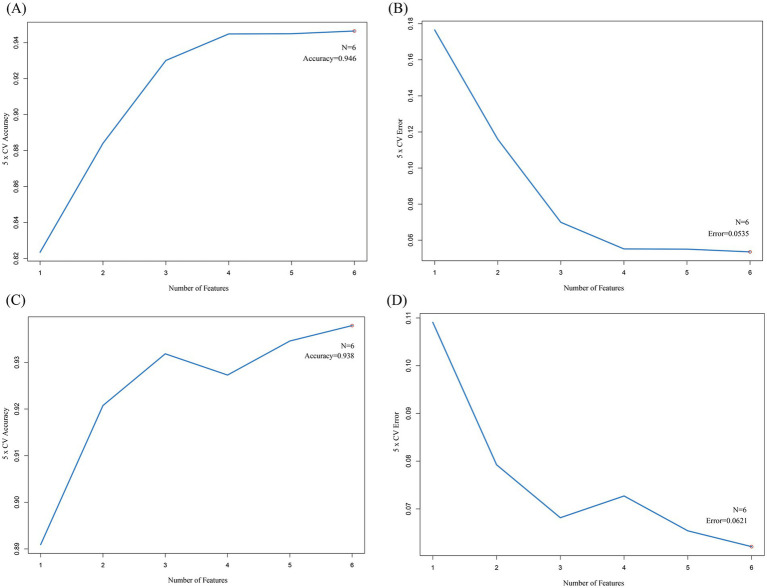
The SVM results. **(A)** This line plot illustrates the relationship between the number of features (genes) and the 5-fold cross-validation (5 × CV) accuracy. The x-axis represents the number of features, ranging from 1 to 6, while the y-axis shows the 5 × CV accuracy. As the number of features increases, the accuracy rises. When the number of features reaches 6 (denoted as “*N* = 6”), the accuracy stabilizes at 0.946. This indicates that using 6 features yields the highest accuracy in the 5-fold cross-validation process for this particular analysis. **(B)** This plot shows the relationship between the number of features and the 5-fold cross-validation error. The x-axis is the number of features, and the y-axis is the 5 × CV error. As the number of features increases from 1 to 6, the error decreases. When the number of features is 6 (*N* = 6), the error reaches a minimum value of 0.0535. This suggests that adding more features reduces the error, and 6 features result in the lowest error rate in the 5-fold cross-validation. **(C)** Similar to Figure **(A)**, this line plot depicts the 5-fold cross-validation accuracy in relation to the number of features. The x-axis lists the number of features, and the y-axis shows the accuracy. The accuracy increases with the number of features, and when the number of features is 6 (*N* = 6), the accuracy is 0.938. This graph further validates the importance of feature number selection for achieving optimal accuracy in the 5-fold cross-validation. **(D)** This plot is analogous to Figure **(B)**, presenting the 5-fold cross-validation error as a function of the number of features. The x-axis is for the number of features, and the y-axis is for the error. As the number of features increases, the error decreases. When the number of features is 6 (*N* = 6), the error is 0.0621. These plots collectively help in determining the optimal number of features to use in a model to achieve the best performance in 5-fold cross-validation, balancing accuracy and error rate.

**Figure 10:**
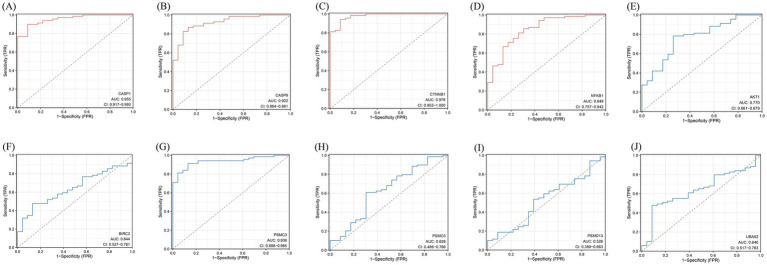
ROC curve. **(A–J)** The abscissa is specific, also known as the false positive rate, and the closer the X-axis is to zero, the higher the accuracy; The ordinate is sensitivity, also known as the true positive rate, and the higher the Y-axis, the better the accuracy. AUC is the area under the ROC curve, which is often used in the evaluation of diagnostic tests, and the value range is generally 0.5-1, and the closer the AUC is to 1, the better the diagnostic effect of this variable in predicting the outcome. The area under the curve (AUC) values and 95% confidence intervals (CI) are shown, indicating the diagnostic performance of these genes in distinguishing relevant conditions. The diagnostic model and the ROC curve in the 10 intersecting genes. Red represents the ROC curve of the upregulated intersecting gene model, and blue represents the ROC curve of the downregulated intersecting gene model.

SVM is commonly used to build diagnostic models in medical imaging and gene expression analysis. For example, in MRI image classification of acute ischemic stroke, SVM achieved an AUC of 0.89 (34). The support vector machine was performed on the 10 upregulated hub cross genes and 10 downregulated hub cross genes to select the genes for the final model. All 10 upregulated genes were subjected to five-fold cross-validation. The results showed the highest accuracy and the lowest error rate when six genes were included (Figures 9A,B). All 10 downregulated genes were subjected to five-fold cross-validation. The results showed the highest accuracy and the lowest error rate when six genes were included (Figures 9C,D). Machine learning models transform high-dimensional gene data into quantifiable diagnostic tools, enhancing the clinical applicability of biomarkers through algorithm optimization. In this study, SVM screened 6 characteristic genes from upregulated and downregulated intersecting genes, respectively, through 5-fold CV (Figures 9A–D), and its nonlinear modeling capability improved the capture of complex gene interaction patterns (Figure 10).

### 3.8 Construction of diagnostic model

**Figure 11:**
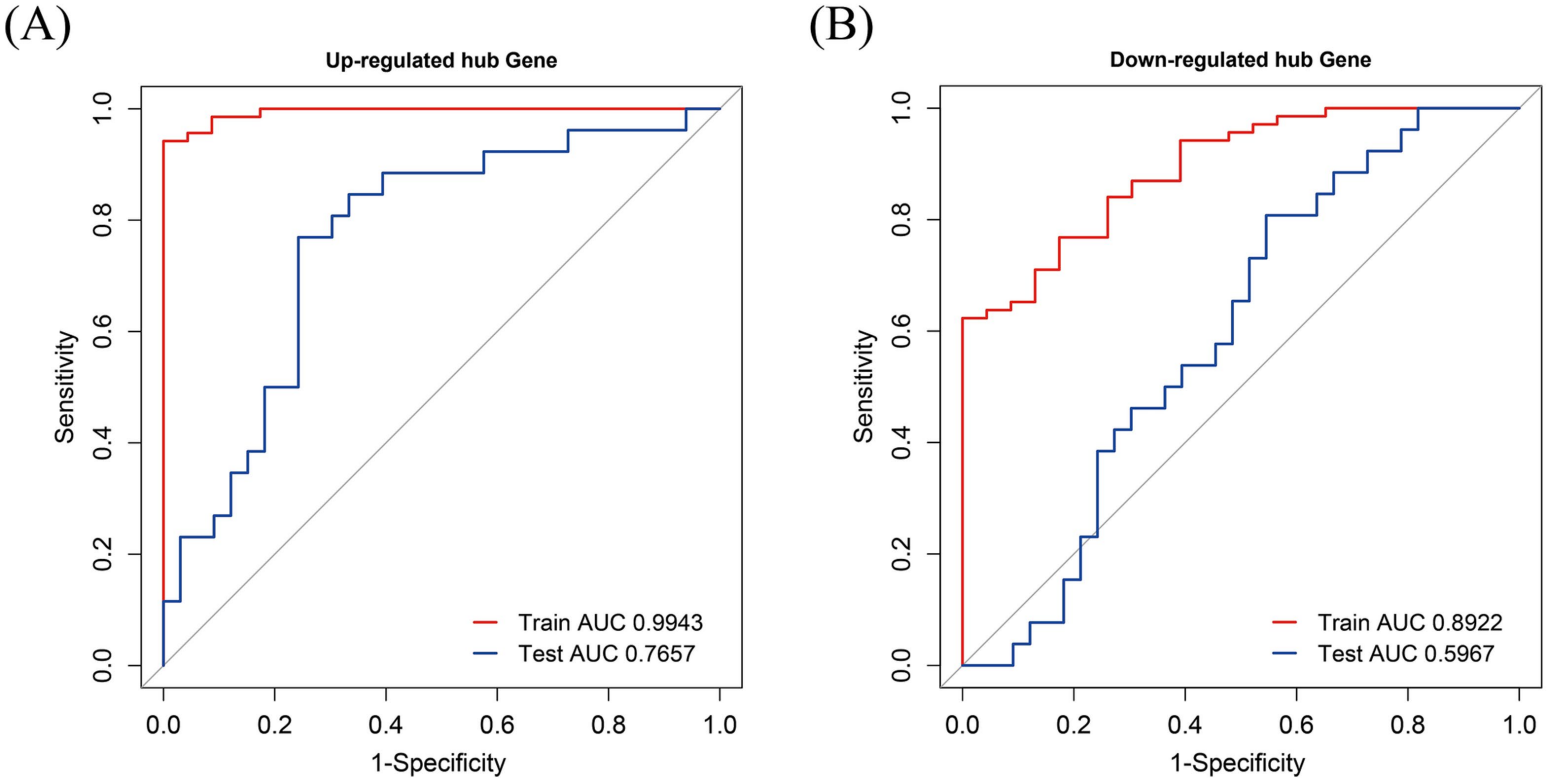
ROC curve. **(A)** Receiver Operating Characteristic (ROC) curve for upregulated hub genes. The x-axis represents 1-Specificity, also known as the false positive rate, and the y-axis represents Sensitivity, which is the true positive rate. The red curve corresponds to the training set, with an Area Under the Curve (AUC) value of 0.9943 (*p*-value<0.0001, 95% CI = 0.9857–1.0000), indicating an extremely high performance of the model in the training phase for predicting the relevant conditions associated with upregulated hub genes. The blue curve represents the test set, having an AUC value of 0.7657 (*p*-value = 0.9998, 95% CI = 0.6402–0.8913). This suggests that while the model still shows good discriminatory ability in the test set, there is a certain gap compared to the training set performance. Overall, this ROC curve provides an assessment of how well the model can distinguish between positive and negative cases for upregulated hub gene. **(B)** ROC curve for downregulated hub genes. Similar to Figure **(A)**, the x-axis is 1-Specificity and the y-axis is Sensitivity. The red curve, representing the training set, has an AUC value of 0.8922 (*p*-value<0.0001, 95% CI = 0.8249–0.9596), showing a relatively high performance of the model during training for down - regulated hub genes. The blue curve, which is for the test set, has an AUC value of 0.5967 (*p*-value = 0.1048, 95% CI = 0.4504–0.7431). This indicates that the model’s ability to discriminate between positive and negative cases in the test set for downregulated hub genes is relatively weaker compared to the training set, and is closer to a random guess (an AUC of 0.5 represents random performance). These ROC curves are crucial for evaluating the effectiveness of the model in predicting the relevant conditions for downregulated hub genes.

**Figure 12:**
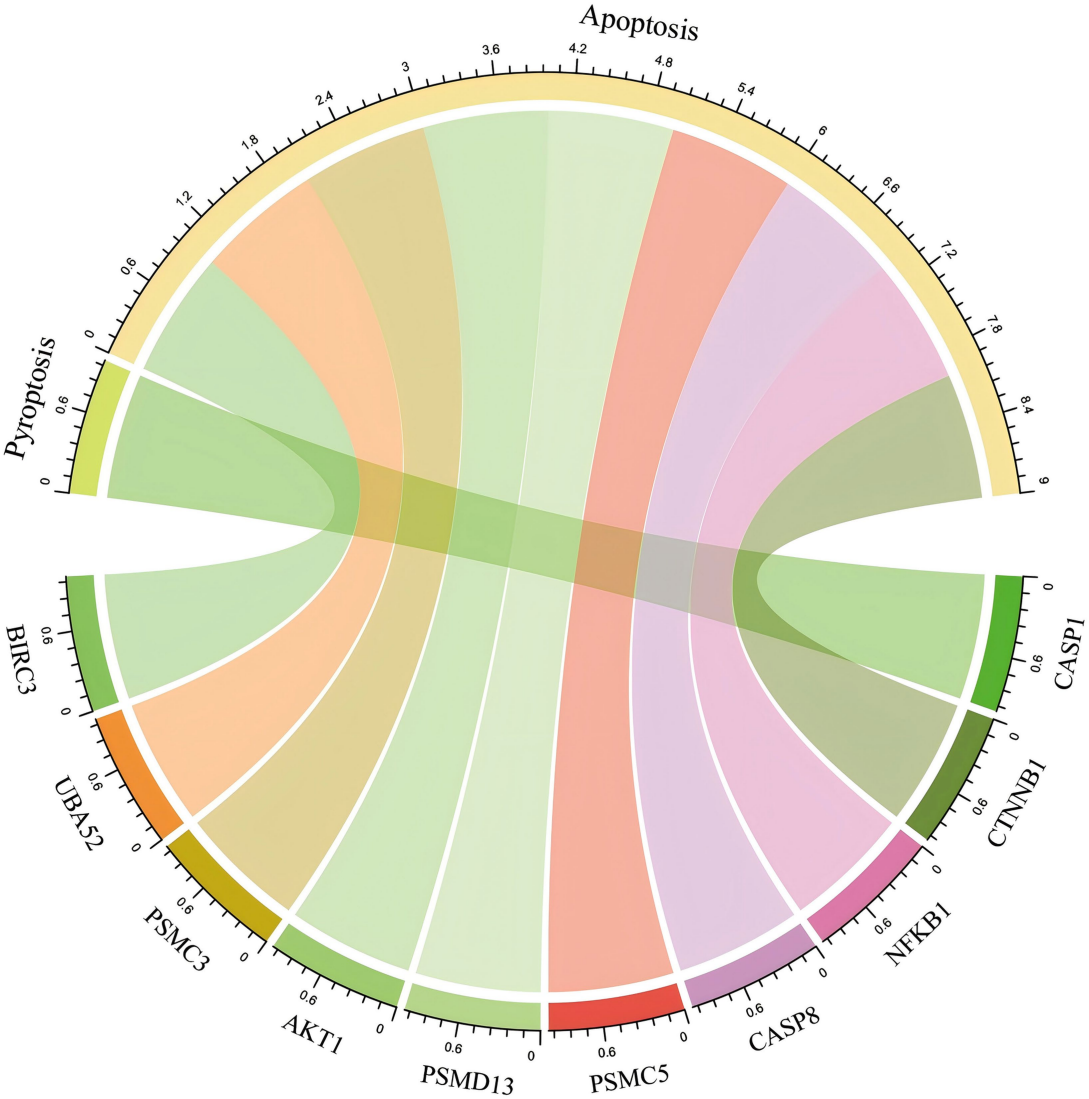
Hub genes that crossed with genes for PANoptosis. The chord diagram shows the distribution of 4 hub upregulated intersecting genes and 6 hub downregulated intersecting genes in the three types of PANoptosis.

The model was established according to the upregulated intersecting genes screened by three machine learning methods and the AUC value of the ROC curve was higher than 0.5. After the validation of the GSE16561 dataset, the AUC value of the ROC curve was 0.7657 (*p*-value = 0.9998) (Figure 11A). The model was established according to the downregulated intersecting genes screened by three machine learning methods and the AUC value of the ROC curve was higher than 0.5. After the validation of the GSE16561 dataset, the AUC value of the ROC curve was 0.5967 (*p*-value = 0.1048) (Figure 11B). Insufficient sample size in the validation set may result in a test power lower than 80%, leading to a *p*-value > 0.05. However, the AUC shows a certain trend (e.g., AUC = 0.7657 in Figure 11A), which can be regarded as a clue for the preliminary exploration of the model’s feasibility. Nevertheless, further optimization and validation are still required. To address the potential false-negative result in the downregulated gene model, we performed a post-hoc power analysis using the pwr package in R (35). Assuming a medium effect size (Cohen’s *d* = 0.2, corresponding to AUC = 0.6) and a target power of 80%, the required sample size for each group (IS patients and controls) was calculated as 160, totaling 320 samples. However, the validation dataset (GSE16561) contained only 63 samples, which is far below the threshold for adequate power. This indicates that the non-significant *p*-value (*p* = 0.1048) is likely attributable to insufficient statistical power rather than the true absence of diagnostic utility for downregulated genes (Supplement 5). The Pearson correlation in SPSS Statistics 21 Software found that these four intersecting genes were significantly correlated with IS (Supplement 2). The results showed that the diagnostic model constructed using some genes had good diagnostic significance. Finally, according to the AUC value of the ROC curve of the screened key genes, one upregulated intersecting gene and three downregulated intersecting genes higher than 0.9 were selected, which were CASP1, CASP8, CTNNB1, and PSMC3 (Supplement 3). In addition, chord diagrams are used to show the relationship between the 10 intersecting genes screened and the three types of PANoptosis. Combining the predictive efficacy of machine learning models (LASSO/RF/SVM), genes with the greatest contribution to IS classification were selected to ensure their clinical diagnostic value (Figure 12).

## 4 Discussion

Abnormal activation of pyroptosis, apoptosis, and necroptosis occurs after IS; however, the mechanisms of these different forms of regulatory death are not independent. In recent years, it has been found that these processes interact with each other, and the concept of ‘PANoptosis’ has been proposed after integrating the three PCDs. However, a correlation between PANoptosis and IS has not yet been established. Therefore, this study aimed to investigate the involvement of PANoptosis in IS.

The 10 downregulated intersecting genes were analyzed by KEGG enrichment (Table 1). Through enrichment analysis, we found that the screened intersecting genes between PANoptosis and downregulated genes were primarily enriched in the apoptosis pathway. Six intersecting genes were expressed, including AKT Serine/Threonine Kinase 1 (AKT1), NF-κB p65 (RELA), myeloid cell leukemia-1 (MCL1), baculoviral IAP repeat containing 3 (BIRC3, also called cIAP2), CASP3 and BCL2 Like 1 (BCL2L1). Apoptosis mainly involves extrinsic and intrinsic pathways. The extrinsic pathway involves transmembrane death receptors of the TNF receptor gene family. The characteristic ligands and their corresponding receptors are FasL/FasR and TNF-*α*/TNFR, respectively. The ligand binds to the receptor to facilitate the signaling pathway and to the adaptor protein FAS-associated protein FADD to transmit the signal. Subsequently, it affects PCD via the action of protease caspases. FADD binds to the CASP8 precursor protein and initiates the execution phase of apoptosis, which in turn leads to a caspase cascade. CASP3 is then activated to degrade intracellular structural and functional proteins, leading to apoptosis. Ubiquitylation of RIPK1 by cellular inhibitors of apoptosis (cIAPs) stabilizes the complex and induces the activation of the transcription factor NFκB (36). One mechanism that has been suggested is the induction of repressive/inactive RelA–NF-κB complexes that mediate apoptosis by actively downregulating NF-κB-dependent, anti-apoptotic gene transcription (37). TRAF1/2 and cIAP1/2 are members of the TNF receptor-associated factor (TRAF) and the inhibitor of apoptosis (IAP) families, respectively. They are critical for both the TNFα-induced canonical and the non-canonical NF-κB signaling pathways (38). The intrinsic pathway activates apoptosis, with the mitochondria as the core. Apoptotic signals, such as DNA damage and abnormal cell signals, can trigger an increase in expression of the proapoptotic protein Bax, or increased expression of BH3 domain-containing proteins that subsequently competitively bind the apoptotic protein Bcl-2/Bcl-xl to release Bax/Bak from inhibition. Free Bax and Bak form oligomers and form pores in the mitochondrial membrane, causing MOMP. In turn induces the release of cytochrome c (Cyt C) from the mitochondria, leading to the formation of apoptotic bodies and the activation of CASP9. Finally, cells undergo apoptosis via protease hydrolysis. Among these, MCL1 is the ortholog of BCL2, and AKT/PKB (protein kinaseB) has an important role in the regulation of NF-kappa-B-dependent gene transcription and positively regulates the activity of CREB1 (39) (cyclic AMP (cAMP)-response element binding protein). The phosphorylation of CREB1 (39) induces the binding of accessory proteins that are necessary for the transcription of pro-survival genes such as BCL2 and MCL1 (Supplementary Figure 1).

**Table 1:** Expression of 10 downregulated intersecting genes in the KEGG pathway.

KEGG pathway	*p* value	Count	Downregulated intersecting genes
Pathways of neurodegeneration - multiple diseases	1.440349e-07	7	PSMC3/RELA/PSMD13/CASP3/UBA52/PSMC5/BCL2L1
Apoptosis	2.359531e-09	6	AKT1/RELA/CASP3/MCL1/BIRC3/BCL2L1
Epstein–Barr virus infection	2.638075e-08	6	PSMC3/AKT1/RELA/PSMD13/CASP3/PSMC5
Parkinson disease	1.481183e-07	6	PSMC3/PSMD13/CASP3/UBA52/PSMC5/BCL2L1
Alzheimer disease	1.295655e-06	6	PSMC3/AKT1/RELA/PSMD13/CASP3/PSMC5

The 10 upregulated intersecting genes were analyzed by KEGG enrichment (Table 2). Through enrichment analysis, we found that the screened intersecting genes between PANoptosis and upregulated genes were mainly enriched in the necroptosis pathway, where a total of seven intersecting genes were expressed. The seven crossover genes were CASP1, CASP8, signal transducer and activator of transcription 3 (STAT3), baculoviral IAP repeat containing 2 (BIRC2, also called cIAP1), TNFRSF1A (TNFR1), FADD, and Toll-like receptor 4 (TLR4). Necroptosis is induced by toll-like receptors, death receptors, interferons, and some other mediators. TNF and cell membrane receptor TNFR1 induce the formation of complex I, which includes TNFR1-related death domain protein (TRADD), RIPK1, and TRAF (40). And TRAF1/2 and cIAP1/2 are members of the TNF receptor associated factor (TRAF) and the inhibitor of apoptosis (IAP) families, respectively. They are critical for both the TNFα-induced canonical and the non-canonical NF-κB signaling pathways (38). And TLR4 initiates the necroptosis mediated by TRIF and RIPK3 (41, 42). Interferon (IFN) and INF-R induce the formation of the JAK–STAT complex (43–45) and involve in necroptotic apoptosis. Additionally, FADD can be activated by the death receptor FAS. Notably, when Caspase-8 is active, it forms a Complex II with RIPK1 and FADD to initiate the execution phase of apoptosis. If Caspase-8 is inhibited, as well as being phosphorylated by RIPK, RIPK3 expression is induced. The complex then transformed into necrotic complexes (RIPK1, RIPK3, mixed lineage kinase domain-like protein (MLKL), etc.), which activate necroptosis (32–34). Phosphorylated MLKL can oligomerize and form pore complexes that translocate cells to the plasma membrane, interact with phosphatidylinositol, and cause membrane permeability and cell destruction. MLKL induces plasma membrane permeability and leads to the spillage of cell contents into organs, resulting in the appearance of inflammatory phenotypes and the release of damage associated molecular patterns (DAMPs). In addition, MLKL signaling activates the NLRP3 inflammasome, which in turn activates CASP1 and triggers the release of the proinflammatory cytokine Interleukin 1 beta (IL-1β). Interestingly, the level of proinflammatory cytokine release was considerably lower than that induced by the TNF*α*-RIPK-MLK-NF-κB pathway. It has been suggested that cell-autonomous inflammatory cytokine expression is coordinated with the release of DAMPs to enhance immune response (46, 47) (Supplementary Figure 2).

**Table 2:** Expression of 10 upregulated intersecting genes in the KEGG pathway.

KEGG pathway	*p* value	Count	Upregulated intersecting genes
Salmonella infection	1.611331e-11	8	CTNNB1/CASP1/CASP8/BIRC2/TNFRSF1A/FADD/TLR4/NFKB1
Toxoplasmosis	4.705485e-12	7	CASP8/STAT3/BIRC2/TNFRSF1A/CASP9/TLR4/NFKB1
Necroptosis	6.088554e-11	7	CASP1/CASP8/STAT3/BIRC2/TNFRSF1A/FADD/TLR4
Hepatitis C	6.088554e-11	7	CTNNB1/CASP8/STAT3/TNFRSF1A/CASP9/FADD/NFKB1
Influenza A	1.062144e-10	7	CASP1/CASP8/TNFRSF1A/CASP9/FADD/TLR4/NFKB1

Based on previous studies, upregulated and downregulated DEGs that crossed with genes for PANoptosis also had the characteristics of apoptosis and pyroptosis. Notably, the overlap between enriched pathways (e.g., TNF signaling, apoptosis) and PANoptosome components (RIPK1, CASP8, NLRP3) suggests that PANoptosis integrates these pathways. For instance, TNF-α activates both necroptosis (via RIPK1/RIPK3) and pyroptosis (via NLRP3 inflammasome), creating a feedforward inflammatory loop in IS. At the end of the study, through the analysis of three machine learning methods, we finally selected four up-regulated and six down-regulated crossover genes. Then, considering the magnitude of the AUC values in the ROC curve, according to the diagnostic prediction model, we included three upregulated crossover genes and one down-regulated crossover gene, namely CASP1, CTNNB1, CASP8, and PSMC3. We explored the involvement of extensive apoptosis in IS and found that both upregulated and downregulated intersecting genes were involved in IS; thus, we analyzed these four intersecting genes separately.

Caspase-1 (CASP1) belongs to pyroptosis. Pyroptosis primarily mediates the activation of multiple caspases, including CASP1, through inflammasomes, resulting in the shearing and multimerization of various gasdermin family members. This causes pores in the cell membrane, resulting in membrane rupture and death. Pyroptosis is also defined as “an inflammasome-dependent cell death and an effector mechanism of the inflammasome” in terms of the inflammatory effects accompanying its occurrence. The activation mechanisms can be divided into CASP1-dependent and CASP1-independent pathways (20). The CASP1-dependent pathway was closely related to our study. Ischemia after IS causes tissue necrosis, which releases molecules (DAMPs) that cause inflammation. Then, the Pattern Recognition Receptors (PRRs) expressed in microglia and macrophages are activated to induce the assembly of inflammasomes (such as NLRP3), promote the activation of CASP1, and then directly lyse Gasdermin D to initiate pyroptosis. In IS, it has been demonstrated that Caspase-1 expression is significantly increased in neurons after IS (48).

CTNNB1, Caspase-8 (CASP8) and PSMC3 belong to apoptosis. Apoptosis, also known as programmed cell death, is a type of regulatory cell death, including marked biochemical and morphological changes. Apoptosis is triggered through two major pathways referred to as the intrinsic and extrinsic pathways (36, 49). Overexpression of Caspase-8 results in apoptosis, and mutation of its catalytic cysteine residue abolishes its apoptotic potential (50). Besides, CTNNB1, CASP8, and PSMC 3 are all actively involved in the process of apoptosis. As a key initiator of extrinsic apoptosis, CASP8 activates the mitochondrial apoptosis pathway by cleaving Bid (14, 25). When its activity is inhibited, it can trigger RIPK3/MLKL-mediated necroptosis. TNF-*α* recruits CASP8 through TNFR1 to form complex II, inducing neuronal apoptosis. If CASP8 is inhibited (e.g., by post-ischemic oxidative stress), the process shifts to necroptosis, exacerbating inflammation (51–53). A study treating ischemic stroke with a monoclonal antibody targeting TNF- α found that the antibody favorably modulates microglial M1 / M2 polarization, rebalances Th 17 / Treg, cell dynamics, and suppresses Caspase-8-mediated GSDMD cleavage to prevent microglial pyroptosis. And provides a promising therapeutic strategy for ischemic stroke (54). CTNNB1 (*β*-catenin) is a core component of the Wnt/β-catenin pathway, regulating cell proliferation and apoptosis. In apoptosis, it inhibits mitochondrial membrane permeability by interacting with Bcl-2 (25). Downregulation of CTNNB1 after cerebral ischemia may inactivate the Wnt pathway, releasing inhibition of apoptosis and exacerbating inflammation through NF-κB activation (22). Some studies have found that CTNNB1 can be a potential drug target for the treatment of ischemic stroke (55). As a subunit of the 26S proteasome, PSMC3 is involved in ubiquitinated protein degradation and regulates the stability of apoptosis-related proteins (e.g., Bcl-2, cIAPs) (21, 25). Downregulation of PSMC3 leads to proteasome dysfunction, accumulation of pro-apoptotic proteins (e.g., Bax), and sustained activation of inflammation by inhibiting degradation of NF-κB negative regulators (21). The PSMC 3 genetic locus was found to be associated with ischemic stroke in a meta-analysis study (56).

Finally, it is worth noting that the diagnostic model was validated on the GSE16561 dataset, which also demonstrated the excellent diagnostic performance of our model. However, this study has limitations. The sample size was relatively small due to the limited number of relevant datasets, necessitating the use of a validation set for result verification. While the upregulated gene model demonstrated robust diagnostic performance, the downregulated gene model’s limited validation performance highlights the need for caution. This may reflect either true biological variability or, more likely, the small sample size and high heterogeneity in the validation dataset. Future studies with larger, clinically annotated cohorts are essential to validate the role of downregulated PANoptosis genes in IS. Second, although we have identified some enriched pathways and key genes, their regulatory processes and interactions have not been elucidated. Studies with larger sample sizes are needed to identify potential biomarkers of PANoptosis associated with IS. The identification of PANoptosis-related gene signatures in IS patients offers a new perspective on neuroinflammation and cell death crosstalk. However, it is critical to interpret these findings with caution. Bioinformatics analyses, while powerful for hypothesis generation, cannot substitute for experimental validation. For instance, the co-expression of CASP1 (pyroptosis) and RIPK3 (necroptosis) in our PPI network suggests PANoptosome formation, but this molecular complex has not been physically detected in ischemic brain tissue. Additionally, the study lacks functional validation—e.g., whether knocking down CASP8 reduces PANoptosis-like cell death in neuronal cultures or mitigates infarct size in animal models. Without such data, the claim of PANoptosis in IS remains correlational. Furthermore, clinical translation is premature, as we did not assess the stability of these biomarkers across different ethnic groups or their utility in distinguishing IS from other stroke subtypes.

## 5 Conclusion

The diagnostic model developed in this study showed excellent performance in both the modeling and validation datasets, indicating that the expression patterns of these genes (co-upregulation or co-downregulation) showed certain specificity in patients with IS. The anomalous expression patterns of PANoptosis-related genes in IS may reflect the mechanism of PANoptosis involved in the regulation of IS pathogenesis. In summary, our study establishes PANoptosis-related genes as robust diagnostic biomarkers for IS and highlights their roles in integrating cell death and inflammation. These findings provide a rationale for developing PANoptosis-targeted therapies to improve IS outcomes, pending validation in preclinical and clinical settings.

## Data Availability

The original contributions presented in the study are included in the article/Supplementary material, further inquiries can be directed to the corresponding authors.

## Footnotes

^1^ http://www.ncbi.nlm.nih.gov/geo/

^2^ https://cn.string-db.org/

## Glossary

IS: Ischemic stroke
DEGs: Differentially expressed genes
GEO: Gene Expression Omnibus
ROC: Receiver operating characteristic curve
AUC: Area under the curve
PCD: Programmed cell death
NLRP3: NOD-like receptor thermal protein domain associated protein 3
RIPK1: Receptor-interacting serine/threonine-protein kinase 1
RIPK3: Receptor-interacting serine/threonine-protein kinase 3
MLKL: Mixed-lineage kinase domain-like
TNF-α: Tumor necrosis factor alpha
ASC: Apoptosis-associated speck-like protein
CASP8: Caspase 8
ZBP1: Z-DNA binding protein 1
BP: Biological process
KFGG: Kyoto Encyclopedia of Genes and Genomes
PPI: Protein-protein interaction
RF: Random Forest
SVM: Support Vector Machine
PCA: Principal Component Analysis
FASLG: Fas ligand
UACA: Uveal autoantigen with coiled-coil domains and ankyrin repeats protein
LY96: Lymphocyte antigen 96
FADD: Fas-associated protein with death domain
TRADD: TNFR1-associated death domain protein
HMGB1: High-mobility group box 1
TNFRSF1A: Tumor necrosis factor receptor superfamily, member 1A
IL1β: Interleukin 1 beta
BAK1: Brassinosteroid insensitive 1-associated receptor kinase 1
DFFB: DNA fragmentation factor subunit beta
DFFA: DNA fragmentation factor subunit alpha
Cyt C: Cytochrome c
BAX: Apoptosis regulator BAX
CASP1: Caspase 1
CTNNB1: catenin beta1
PSMC3: Proteasome 26S Subunit, ATPase 3
CASP6: Caspase 6
CASP9: Caspase 9
MLKL: Mixed lineage kinase domain-like protein
DAMPs: damage associated molecular patterns
GSDMD: Gasdermin-D
UACA: Uveal autoantigen with coiled-coil domains and ankyrin repeats
TNFR1: Tumor Necrosis Factor Receptor 1
TNFR2: Tumor Necrosis Factor Receptor 2
PRRs: Pattern Recognition Receptors
AKT1: AKT Serine/Threonine Kinase 1
AKT/PKB: protein kinase B
RELA: NF-κB p65
MCL1: Myeloid Cell Leukemia-1
BIRC3: Baculoviral IAP Repeat Containing 3
BIRC2: Baculoviral IAP Repeat Containing 2
BCL2L1: BCL2 Like 1
BCL2: B-cell lymphoma-2
cIAP1/2: Cellular inhibitors of apoptosis proteins 1/2
TRAF: TNF receptor-associated factor
MOMP: mitochondrial outer membrane permeabilization
CREB1: cyclic AMP (cAMP)-response element binding protein
STAT: Signal Transducers and Activators of Transcription
STAT3: signal transducer and activator of transcription 3
TLR4: Toll-like receptor 4
JAK: Janus Kinase
