# Supplementary material for: Identification of PANoptosis-related genes as biomarkers in ischemic stroke
Source: Front Neurol. 2025 Jul 25;16:1560514. doi: 10.3389/fneur.2025.1560514 (PMC12333936; doi:10.3389/fneur.2025.1560514)
Supplement: Supplementary file 1 [file Table_1.docx]

Supplementary Figures

Identification of PANoptosis-related genes as biomarkers in ischemic stroke

# Supplementary Figures


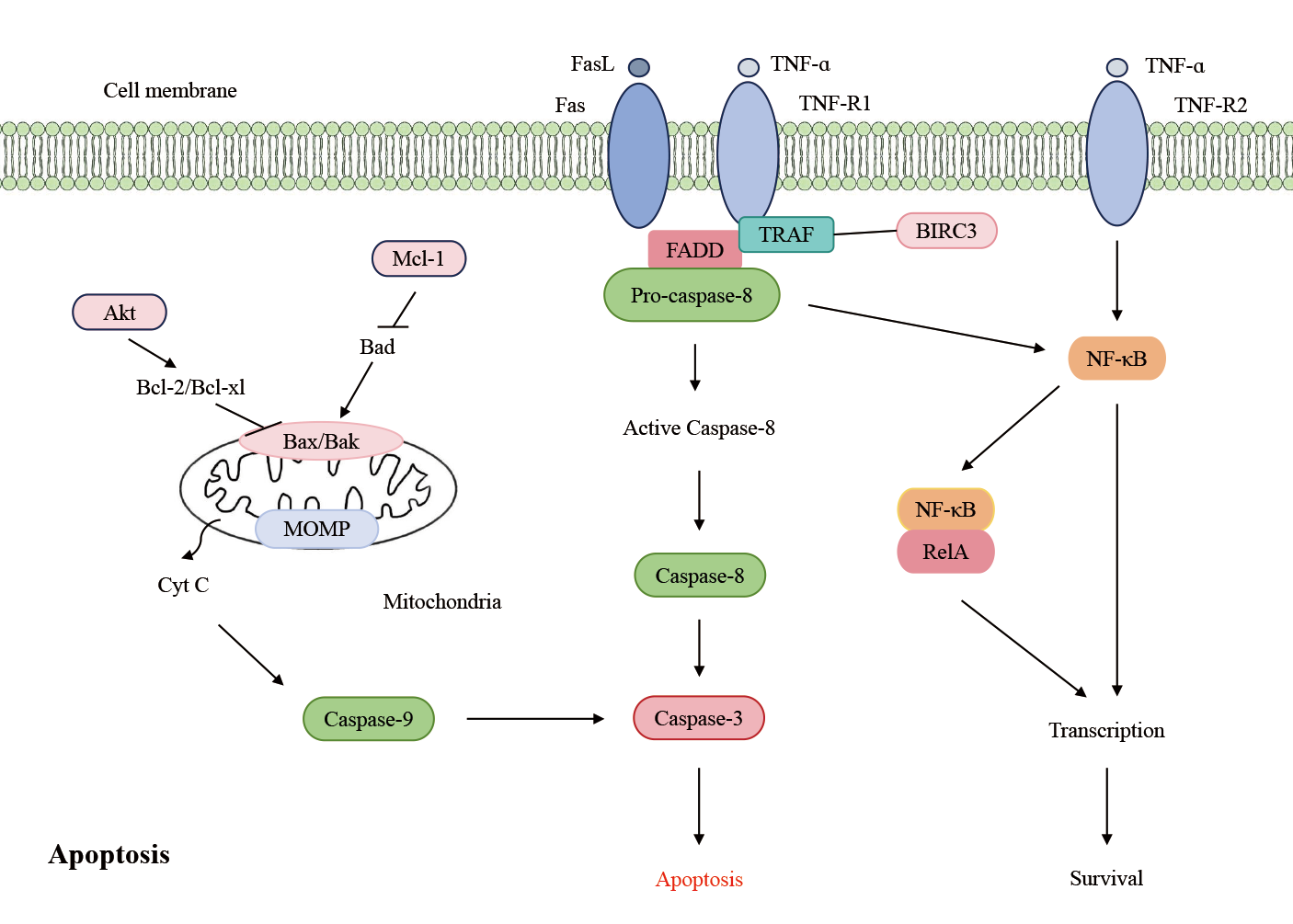


**Supplementary Figure 1.** | **Apoptosis pathway.** The extrinsic pathway is initiated by the combination of FasL and TNF-α with death receptors (FasR and TNFR), and combine with FADD to transmit signals. FADD binds to the procaspase-8 and initiates the execution phase of apoptosis. After activation of caspase-8 is then activated to degrade intracellular structural and functional proteins, leading to apoptosis. The intrinsic pathway of apoptosis is initiated by the cell itself, and caspase 3 is finally activated to cause apoptosis. cIAP2(BIRC3), Cellular inhibitors of apoptosis proteins 2; AKT/PKB, protein kinase B; MCL1, Myeloid cell leukemia‐1; BCL2, B-cell lymphoma-2; RelA, NF-κB p65; MOMP, mitochondrial outer membrane permeabilization.


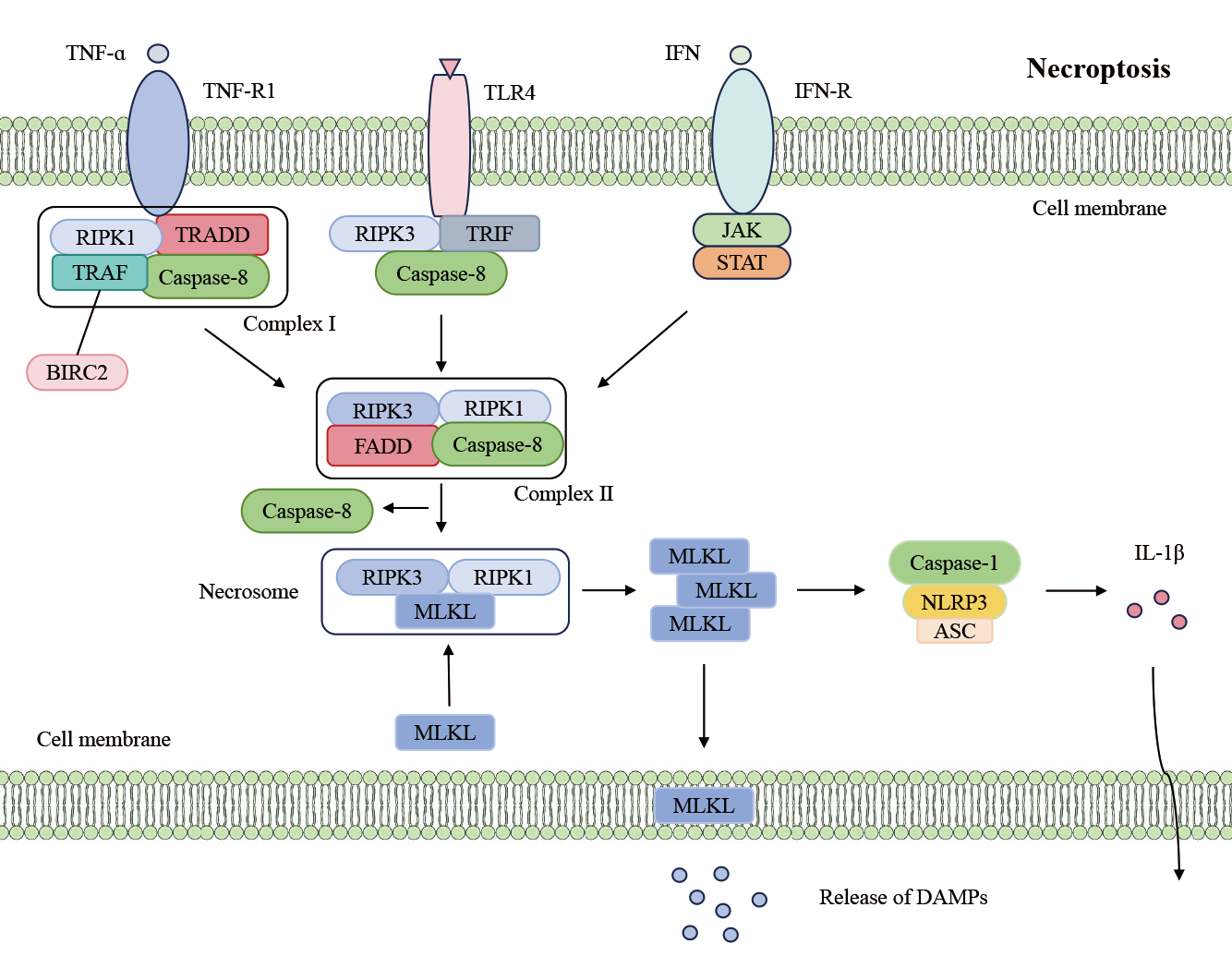


**Supplementary Figure 2.** | **Necroptosis pathway.** Necroptosis is triggered mainly by extracellular stimuli. TNF-α/INF binds to TNFR1/INF-R and induces the formation of complexes. TLR4 initiates the necroptosis mediated by TRIF and RIPK3. Complex I can also be released and recruit FADD to form complex II. Complex II can mediate the activation of caspase-8, leading to apoptosis. If caspase-8 is deficient, RIP1, RIP3, and MLKL form necrosome. MLKL induces plasma membrane permeability and leads to the spillage of cell contents into organs, resulting in the appearance of inflammatory phenotypes and the release of DAMPs. In addition, MLKL signaling activates the NLRP3 inflammasome, which in turn activates CASP1 and triggers the release of the proinflammatory cytokine IL-1β. TRADD, TNFR1-associated death domain protein; cIAP1(BIRC2), Cellular inhibitors of apoptosis proteins 1; JAK, Janus Kinase; STAT, Signal Transducers and Activators of Transcription; TLR4, Toll-like receptor 4.
