# Supplementary material for: Identification of PANoptosis-related genes as biomarkers in ischemic stroke
Source: Front Neurol. 2025 Jul 25;16:1560514. doi: 10.3389/fneur.2025.1560514 (PMC12333936; doi:10.3389/fneur.2025.1560514)
Supplement: Supplementary file 2 [file Table_2.docx]

Supplementary Tables

Identification of PANoptosis-related genes as biomarkers in ischemic stroke

# Supplementary Tables

Table 1. Expression of 10 downregulated cross genes in the KEGG pathway

| **KEGG pathway** | **P. value** | **Count** | **Downregulated cross genes** |
| --- | --- | --- | --- |
| Pathways of neurodegeneration - multiple diseases | 1.440349e-07 | 7 | PSMC3/RELA/PSMD13/CASP3/UBA52/PSMC5/BCL2L1 |
| Apoptosis | 2.359531e-09 | 6 | AKT1/RELA/CASP3/MCL1/BIRC3/BCL2L1 |
| Epstein-Barr virus infection | 2.638075e-08 | 6 | PSMC3/AKT1/RELA/PSMD13/CASP3/PSMC5 |
| Parkinson disease | 1.481183e-07 | 6 | PSMC3/PSMD13/CASP3/UBA52/PSMC5/BCL2L1 |
| Alzheimer disease | 1.295655e-06 | 6 | PSMC3/AKT1/RELA/PSMD13/CASP3/PSMC5 |

Table 2. Expression of 10 upregulated cross genes in the KEGG pathway

| **KEGG pathway** | **P. value** | **Count** | **Upregulated cross genes** |
| --- | --- | --- | --- |
| Salmonella infection | 1.611331e-11 | 8 | CTNNB1/CASP1/CASP8/BIRC2/TNFRSF1A/FADD/TLR4/NFKB1 |
| Toxoplasmosis | 4.705485e-12 | 7 | CASP8/STAT3/BIRC2/TNFRSF1A/CASP9/TLR4/NFKB1 |
| Necroptosis | 6.088554e-11 | 7 | CASP1/CASP8/STAT3/BIRC2/TNFRSF1A/FADD/TLR4 |
| Hepatitis C | 6.088554e-11 | 7 | CTNNB1/CASP8/STAT3/TNFRSF1A/CASP9/FADD/NFKB1 |
| Influenza A | 1.062144e-10 | 7 | CASP1/CASP8/TNFRSF1A/CASP9/FADD/TLR4/NFKB1 |
