# Supplementary material for: Identification of PANoptosis-related genes as biomarkers in ischemic stroke
Source: Front Neurol. 2025 Jul 25;16:1560514. doi: 10.3389/fneur.2025.1560514 (PMC12333936; doi:10.3389/fneur.2025.1560514)
Supplement: Supplementary file 3 [file Table_3.docx]

Supplementary Material

Identification of PANoptosis-related genes as biomarkers in ischemic stroke

# Supplementary Data

Supplement 1. PANoptosis gene list

| **PANoptosis**  **(Programmed cell death)** | **Gene list** |
| --- | --- |
| **Pyroptosis** | BAK1、TP63、CHMP2B、BAX、GZMB、CHMP4B、GSDMD、GSDME、IL1A、CHMP3、IRF1、IL1B、CHMP2A、CASP1、CASP5、TP53、CHMP7、IL18、CASP3、CHMP4C、IRF2、CYCS、CHMP6、HMGB1、CASP4、ELANE、CHMP4A |
| **Apoptosis** | DBNL、TRAF2、PSMA2、TLR4、ACIN1、STK24、PSMA4、MTOR、H1-3、ROCK1、ITGA5、PRKAR1B、DFFB、BID、IRAK1、AIFM1、CRYBA1、DYNLL2、PSMD1、PSMD9、PRKAR2B、NTRK1、CLSPN、YWHAH、APIP、H1-5、BMF、STK11、TICAM1、CHP1、PSMC5、EXOG、C1QBP、DFFA、UBB、IKBKG、TFDP2、GSN、MYD88、FNTA、PKP1、PPP1R13B、YWHAG、CASP9、MAP3K7、CEACAM6、DSP、UNC5B、LMNB1、ITGB1、CASP6、BBC3、TLE1、UNC5A、NGF、CHP2、PSMC4、PSMB2、PRKCQ、VIM、PPP3CB、PSMD13、PSMD8、PRKAR1A、BCL2L11、PSMA3、IRAK2、CFLAR、PSMA1、AKT1、NTRK2、STK26、PIK3R1、CHUK、PIK3R5、TNFRSF1A、PTRH2、STAT3、MAPK1、PSMB11、PRKACA、PSMB7、XIAP、APAF1、NMT1、TJP2、H1-0、PRKX、PPP3R1、OCLN、SEM1、PPP3CA、PSMD7、PIK3CG、IRAK3、CTNNB1、AKT2、NFKB1、PPP3R2、IKBKB、PRKAR2A、TFDP1、PSMC2、BAD、UACA、BRMS1、PSMC3、CAPN2、IL3RA、PIK3CA、BMX、CASP10、ANKRD13C、ADD1、PSMD2、PSMA8、PSMD12、ATM、YWHAE、DAPK3、PSMB3、DAPK2、DNM1L、CEACAM5、PSMB1、CDH1、NFKBIA、PTK2、PSMA6、TNFSF10、H1-1、YWHAQ、DCC、DIABLO、SFN、TICAM2、BCL2、IL1RAP、TNFRSF10A、PSMB6、LMNA、TJP1、CASP7、MAP3K14、KPNB1、PRKACB、TNFRSF10C、PSMD6、PSMD3、PIK3CB、DSG1、ENDOD1、PSME3、APC、AVEN、DYNLL1、CARD8、PSMB8、PSMD11、PIK3CD、PSME2、CDKN2A、IL3、MAPK8、UBA52、MAGED1、BCAP31、RPS27A、CAV1、BCL2L1、IRAK4、BIRC2、CAPN1、E2F1、PIK3R2、MAPK3、OMA1、SEPTIN4、PSMA7、CASP8、ARHGAP10、TSC2、PSMB10、SNAI2、ENDOG、PSMB4、PLEC、PMAIP1、SPTAN1、APPL1、PSMB5、PSMD10、NOTCH1、DSG2、LY96、AKT3、DAPK1、SIK1、IL1R1、CHEK2、TNFRSF10B、PDK4、MCL1、PSMA5、MYBBP1A、KPNA1、RELA、TNFRSF10D、PSMC1、TP73、PRKCD、PRKACG、TLE5、MAPT、PSME4、HMGB2、SATB1、TP53BP2、PAK2、H1-4、CSF2RB、PSMD4、GAS2、PSME1、PSMD14、TRADD、SRC、PSMC6、H1-2、DSG3、PSMD5、UBC、PSMB9、PPP3CC、PSMF1、ZNF304、YWHAB、PIK3R3、BIRC3、YWHAZ、CD14、OPA1 |
| **Necroptosis** | RIPK3、MLKL、FAS、FASLG、TLR3、TNF、RIPK1、FADD |

Supplement 2. Pearson correlation between the top 4 intersecting genes of upregulated and IS

| **Upregulated Intersecting genes** | **CASP1** | **CASP8** | **CTNNB1** | **NFKB1** |
| --- | --- | --- | --- | --- |
| **Pearson correlation** | 0.716 | 0.665 | 0.754 | 0.533 |
| **P** | 0.000 | 0.000 | 0.000 | 0.000 |

Supplement 3. Pearson correlation between the top 6 intersecting genes of downregulated and IS

| **Downregulated Intersecting genes** | **AKT1** | **BIRC3** | **PSMC3** | **PSMC5** | **PSMD13** | **UBA52** |
| --- | --- | --- | --- | --- | --- | --- |
| **Pearson correlation** | -0.407 | -0.198 | -0.635 | 0.211 | 0.073 | -0.184 |
| **P** | 0.000 | 0.058 | 0.000 | 0.044 | 0.491 | 0.079 |

Supplement 4. Area under ROC curve (AUC) and 95% confidence interval (95% CI) of 10 intersecting genes.

| **Intersecting genes** | **AKT1** | **BIRC3** | **PSMC3** | **PSMC5** | **PSMD13** | **UBA52** | **CASP1** | **CASP8** | **CTNNB1** | **NFKB1** |
| --- | --- | --- | --- | --- | --- | --- | --- | --- | --- | --- |
| **AUC** | 0.770 | 0.664 | 0.936 | 0.626 | 0.526 | 0.640 | 0.955 | 0.922 | 0.978 | 0.849 |
| **95% CI** | 0.661-0.879 | 0.527-0.761 | 0.888-0.985 | 0.486-0.766 | 0.389-0.663 | 0.517-0.763 | 0.917-0.993 | 0.864-0.981 | 0.953-1.000 | 0.757-0.942 |

# Supplement 5. Sample information on the X axis in Figure 2B

| **Sample information** |
| --- |
| GSM1406033、GSM1406034、GSM1406035、GSM1406036、GSM1406037、GSM1406038、GSM1406039、GSM1406040、GSM1406041、GSM1406042、GSM1406043、GSM1406044、GSM1406045、GSM1406046、GSM1406047、GSM1406048、GSM1406049、GSM1406050、GSM1406051、GSM1406052、GSM1406053、GSM1406054、GSM1406055、GSM1406056、GSM1406057、GSM1406058、GSM1406059、GSM1406060、GSM1406061、GSM1406062、GSM1406063、GSM1406064、GSM1406065、GSM1406066、GSM1406067、GSM1406068、GSM1406069、GSM1406070、GSM1406071、GSM1406072、GSM1406073、GSM1406074、GSM1406075、GSM1406076、GSM1406077、GSM1406078、GSM1406079、GSM1406080、GSM1406081、GSM1406082、GSM1406083、GSM1406084、GSM1406085、GSM1406086、GSM1406087、GSM1406088、GSM1406089、GSM1406090、GSM1406091、GSM1406092、GSM1406093、GSM1406094、GSM1406095、GSM1406096、GSM1406097、GSM1406098、GSM1406099、GSM1406100、GSM1406101、GSM1406102、GSM1406103、GSM1406104、GSM1406105、GSM1406106、GSM1406107、GSM1406108、GSM1406109、GSM1406110、GSM1406111、GSM1406112、GSM1406113、GSM1406114、GSM1406115、GSM1406116、GSM1406117、GSM1406118、GSM1406119、GSM1406120、GSM1406121、GSM1406122、GSM1406123、GSM1406124 |

Supplement 6. The result of Post-hoc Power Analysis

To assess the statistical power of the validation cohort for the downregulated gene model, we used Cohen’s d to quantify the effect size, where d = 2*AUC - 1. For an AUC of 0.5967, d = 0.1934 (approximating a small-to-medium effect). Using the pwr.norm.test function in R, we determined that a sample size of 160 per group (320 total) was required to achieve 80% power at α = 0.05. The validation dataset (GSE16561) included 63 samples, resulting in a power estimate of ~20%, which is insufficient to detect the observed effect.

| **Parameters** | **Results** |
| --- | --- |
| Effect size | Cohen's d=0.1934 |
| α level | 0.05 |
| Power | 0.08 |
| Sample size (per group) | 209.8426 |

Supplement 7. Parameters of LASSO/RF/SVM

1.LASSO Regression

• type.measure = "mse": Uses mean squared error (MSE) as the cross-validation metric. While MSE is typical for regression tasks, it is used here for binary classification due to compatibility with cv.glmnet.

• nfolds = 5: Performs 5-fold cross-validation to balance computational efficiency and bias-variance tradeoff.

• alpha = 1: Enforces pure L1 regularization (LASSO), which promotes sparse feature selection by shrinking irrelevant coefficients to zero.

• nlambda = 100: Generates 100 lambda values logarithmically spaced from the largest value yielding all-zero coefficients to a minimum value

2.Random Forest (RF)

• method = "LGOCV":Specifies Leave Group Out Cross-Validation (LGOCV). If no groups are explicitly defined (via the groups parameter), caret defaults to Leave One Out Cross-Validation (LOOCV).

• number = 10: Uses 10-fold cross-validation to evaluate model performance.

metric = "Accuracy":Uses classification accuracy as the performance metric.

• sizes = 1:(length(candidate.gene)-2): Evaluates models with feature subsets ranging from 1 to ncol(candidate.gene)-2.

3.Support Vector Machine (SVM)

• k = 5: Performs 5-fold cross-validation during feature selection.

halve.above = 100: Halves the number of features at each iteration when the number of features exceeds 100, accelerating feature elimination for high-dimensional data.

• 1:6 in FeatSweep.wrap: Evaluates SVM models using the top 1 to 6 features selected by svmRFE.

*•* Default Kernel and Hyperparameters: The code uses default SVM parameters (e.g., radial kernel with gamma = 1/ncol (x) and cost = 1).
